# Supplementary material for: Differential Expression of miRNAs and Behavioral Change in the Cuprizone-Induced Demyelination Mouse Model
Source: Int J Mol Sci. 2020 Jan 18;21(2):646. doi: 10.3390/ijms21020646 (PMC7014274; doi:10.3390/ijms21020646)
Supplement: Supplementary file 1 [file ijms-21-00646-s001.pdf]

International Journal of
Molecular Sciences

MDPI

Article

Differential Expression of miRNAs and Behavioral Change in the Cuprizone-Induced Demyelination Mouse Model

Seung Ro Han [1,2,†], Yun Hee Kang [1,2,†], Hyungtaek Jeon [2], Suhyuk Lee [2], Sang-Jin Park [3], Dae-Yong Song [3], Sun Seek Min [4], Seung-Min Yoo [1,2], Myung-Shin Lee [1,2,*] and Seung-Hoon Lee [1,5,*]

[1] Eulji Biomedical Science Research Institute, Eulji University School of Medicine, Daejeon 34824, Korea; srhan@eulji.ac.kr (S.R.H.); yhkang@eulji.ac.kr (Y.H.K.); smyoo@eulji.ac.kr (S.-M.Y.)
[2] Department of Microbiology and Immunology, Eulji University School of Medicine, Daejeon 34824, Korea; jhtjoy0405@gmail.com (H.J.); simpson214@naver.com (S.L.)
[3] Department of Anatomy and Neuroscience, Eulji University School of Medicine, Daejeon 34824, Korea; bright11star@naver.com (S.-J.P.); dysong@eulji.ac.kr (D.-Y.S.)
[4] Department of Physiology and Biophysics, Eulji University School of Medicine, Daejeon 34824, Korea; ssmin@eulji.ac.kr
[5] Department of Neurosurgery, Eulji University School of Medicine, Daejeon 34824, Korea
* Correspondence: mslee@eulji.ac.kr (M.-S.L.); nslsh@eulji.ac.kr (S.-H.L.); Tel.: +82-42-259-1662 (M.-S.L.); +82-42-259-1600 (S.-H.L.)
† Both authors contributed equally to this work.

Supplementary Figures and Table

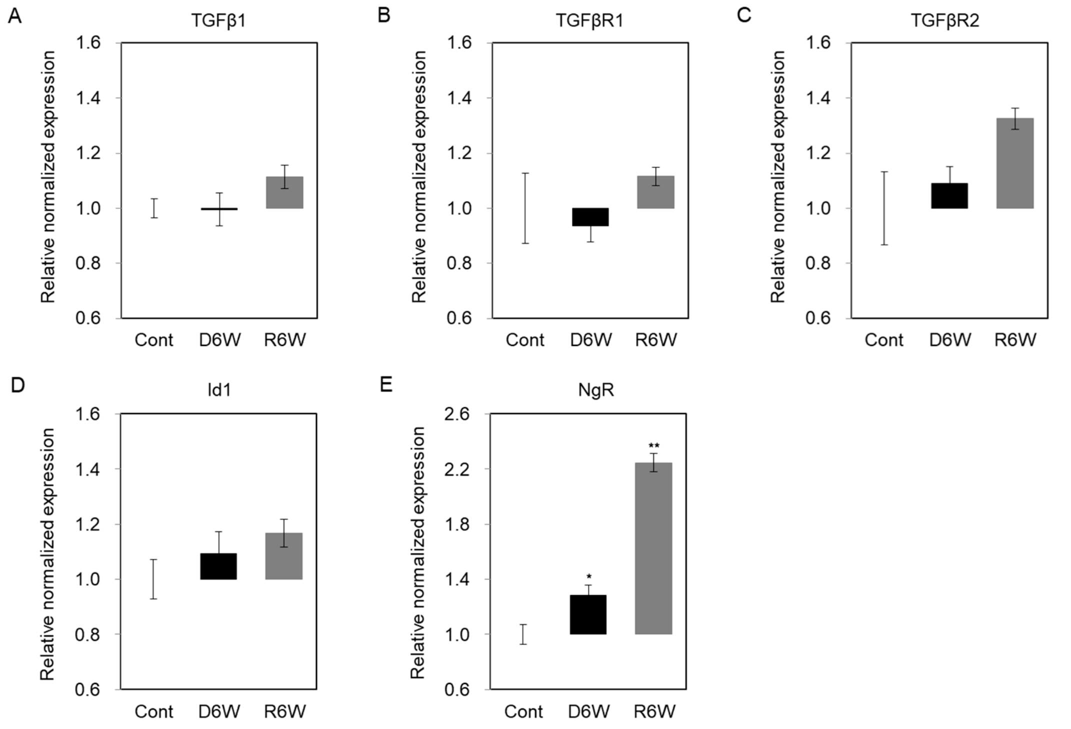

Figure S1. Analysis of miRNA target gene expression by qPCR. (**A**–**D**) The expression levels of TGFβ1, TGFβR1, TGFβR1 and Id1 were not significantly altered in demyelinated mice (D6W) compared to control mice (Cont) by real-time PCR analysis. (**E**) The expression level of NgR was significantly upregulated in D6W compared to Cont. The differences between groups are expressed as $*p < 0.05$, $**p < 0.01$.

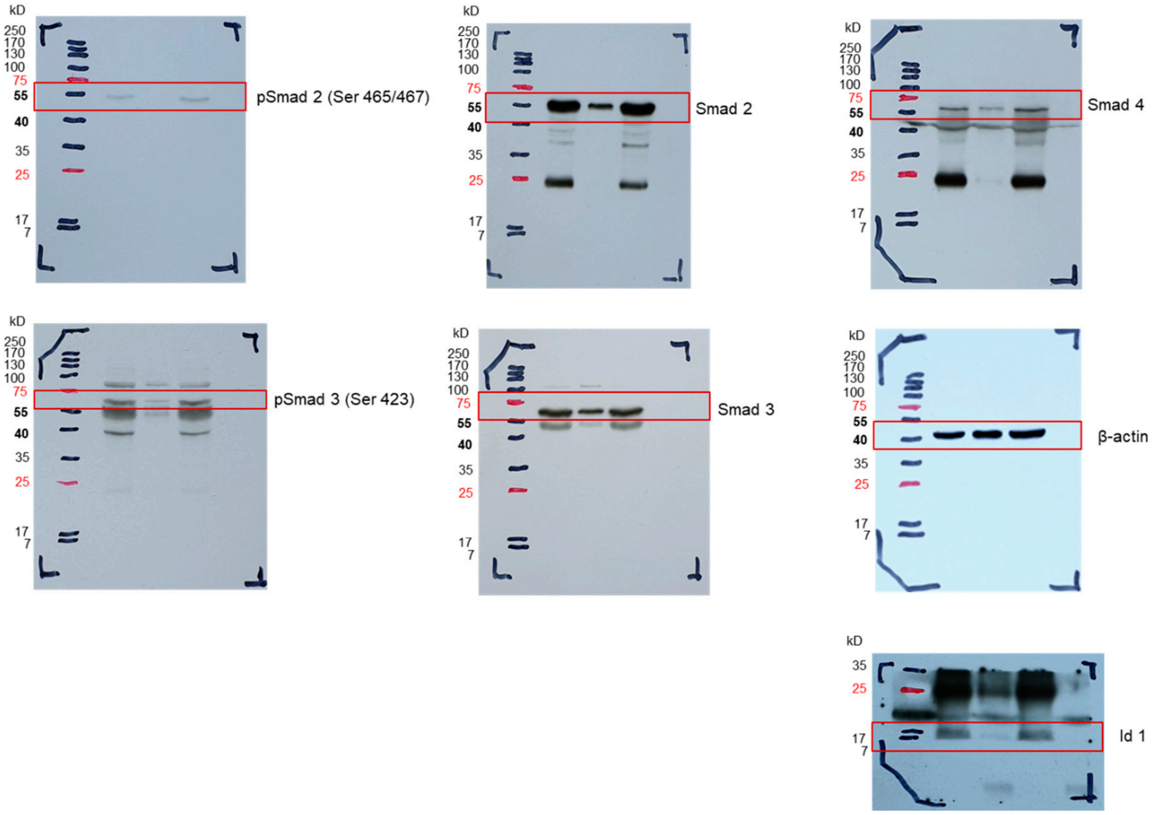

Figure S2. The uncut original western blot images of Figure 8A.

Table S1. The quantitative PCR primers of miRNAs and target genes.

Oligo Name	Length (mer)	Sequence (5' – 3')
**Upregulated miRNA**		
mmu-miR-146a-5p	22	TGAGAACTGAATTCCATGGGTT
mmu-miR-20a-5p	23	TAAAGTGCTTATAGTGCAGGTAG
mmu-miR-155-5p	23	TTAATGCTAATTGTGATAGGGGT
**Downregulated miRNA**		
mmu-miR-145a-5p	23	GTCCAGTTTTCCCAGGAATCCCT
mmu-miR-219a-2-3p	22	AGAATTGTGGCTGGACATCTGT
mmu-miR-338-5p	22	AACAATATCCTGGTGCTGAGTG
**Predicted taget genes**		
TGFβ1	21	CTATGACAAGTTCAAGCAGAG
TGFβR1	20	GTGAAGCCTTGAGAGTAATG
TGFβR2	18	AATATAACACCAGCAATC
Id1	18	TGTTACTCACGCCTCAAG
NgR	18	ACGGCAACCGCATCTCCA
GAPDH	18	GGTATCGTGGAAGGACTC

© 2020 by the authors. Licensee MDPI, Basel, Switzerland. This article is an open access article distributed under the terms and conditions of the Creative Commons Attribution (CC BY) license (http://creativecommons.org/licenses/by/4.0/).
